# Experimental and computational analysis of hybrid fiber metal laminates for vibration behavior in marine structural applications

**DOI:** 10.1038/s41598-024-80961-7

**Published:** 2024-12-02

**Authors:** Anand Pai, Marcos Rodriguez-Millan, Kwong Ming Tse, Sriharsha Hegde, Chandrakant R. Kini, Satish B. Shenoy

**Affiliations:** 1 Department of Aeronautical and Automobile Engineering, Manipal Institute of Technology, Manipal Academy of Higher Education, Manipal, Karnataka 576104 India; 2 Department of Mechanical Engineering, Universidad Carlos III de Madrid, ROR, Avenida de la Universidad, 30 (edificio Sabatini), 28911 Leganés, Madrid Spain; 3 Department of Mechanical and Product Design Engineering, School of Engineering, Swinburne University of Technology, 427-451, Burwood Rd, Hawthorn, VIC 3122 Australia

**Keywords:** Pressure Hulls, Fiber-metal laminates, Vibration damping, Frequency response functions, Harmonic analysis, Engineering, Materials science, Mathematics and computing

## Abstract

Structural advancements in underwater vehicle design necessitate lightweight materials, driving interest in Fiber Metal Laminates (FMLs), known for their high specific strength, stiffness, and corrosion resistance. This study investigates the vibration response of FMLs through combined experimental and numerical analyses, specifically evaluating the novel effects of layerwise acoustic impedance matching on vibration damping within the 0-500 Hz frequency range, which aligns with ocean current frequencies. Various FML stackup sequences were characterized through ASTM E756-05 compliant experiments and ANSYS Harmonic Response simulations. Notably, the introduction of paperboard-epoxy ply results in a rightward shift in natural frequencies, while the exclusion of the metallic face ply leads to a leftward shift across different stackups. Moderate agreement between experimental and numerical results for material modulus highlights the robustness of our findings. Overall, this study provides valuable insights for leveraging FMLs in submersible hulls, underscoring their potential for enhanced vibration-damping characteristics in marine environments.

## Introduction

**Fig. 1:**
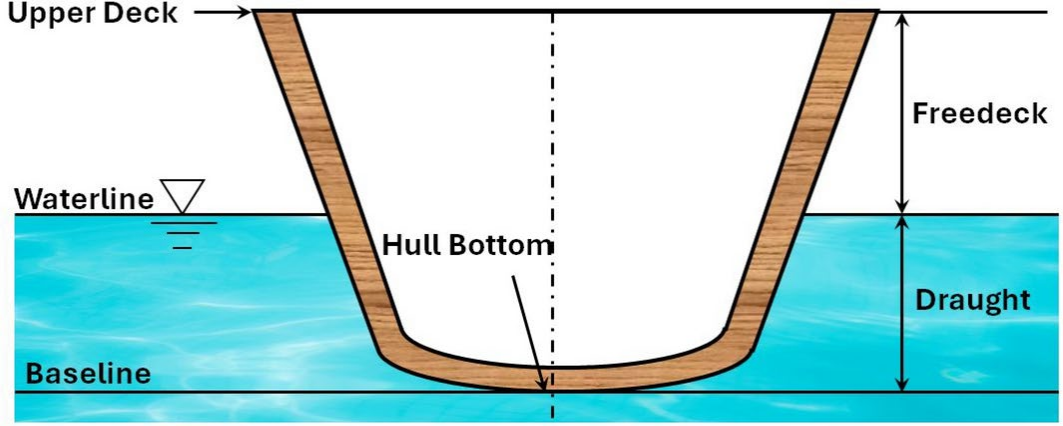
Schematic of a ship hull and terminologies.

Submersibles and underwater vehicles require pressure hulls that possess high hydrospace buoyancy factors. Hence, the use of lightweight materials has become essential to achieve this^1–3^. These structures must meet criteria for yielding, buckling, high strength under various hydrostatic pressures, impact strength, corrosion resistance, and vibration damping. Hydrodynamic loads and oscillations caused by ocean currents, surface winds, and convection also play a role^4,5^. In the early 2000s, composite pressure hulls gained popularity due to the tailored properties of fiber-reinforced composite laminates, by managing the ply orientations^6^. Ship hulls as illustrated in Fig. 1 undergo complex operational mechanics, experiencing hogging and sagging while navigating through ocean currents, resulting in alternating bending moments and low-frequency ship vibrations, with peak ocean current frequencies reaching 250 Hz^7–9^. Ship hull vibrations can also be caused by marine propulsion equipment, systems, and propeller excitations, with elastic waves traveling through the hulls in various forms- flexural, longitudinal and torsion waves^10^. Vibrations originating at the water-hull surface beneath the waterline propagate throughout the ship’s structure. Waves impacting the ship induce, vibrations in the freeboard area, creating dynamic forces on the hull that may be felt on the upper deck. Resonance can intensify vibrations if the wave frequency matches the natural frequency of the ship hulls, a concern addressed by naval architects during design to minimize resonance risk. Under specific conditions, water flow around submerged hull parts can lead to vortex shedding, causing vibrations, more common in slender structures, potentially affecting the draught region^11^. Vibrations in freeboard and draught areas are interconnected, propagating through the entire ship structure and influencing different hull parts^12^. Vibration analysis plays a crucial role in identifying and addressing these potential issues in ship hull structures. By analyzing vibrations, various areas of concern can be identified, including excessive deflection, stress concentration, and fatigue failure. These issues have the potential to cause structural damage or even catastrophic failures. Therefore, vibration analysis is essential for ensuring the structural integrity of the ship^13–15^. Furthermore, excessive vibrations can have a negative impact on the comfort and safety of crew members and passengers on board. High levels of ship vibrations can lead to discomfort, fatigue, and even injuries among the crew. By conducting vibration analysis and implementing appropriate mitigation measures, the comfort and safety levels on the ship can be significantly improved. Vibrations also have implications for the performance and efficiency of the ship. Excessive vibrations can increase fuel consumption, reduce the efficiency of the propulsion system, and diminish the overall operational effectiveness of the ship. Through vibration analysis, ship designs, materials, and systems can be optimized to minimize vibrations and enhance performance and efficiency^16,17^. In addition to their impact on structural integrity, comfort, and performance, vibrations in ship hull structures can generate unwanted noise and affect the ship’s acoustic signature. Excessive noise can have various undesirable consequences, including environmental impacts, regulatory non-compliance, and negative effects on human health. Vibration analysis helps in identifying the sources of noise and enables the development of effective noise control measures, ensuring compliance with regulations and promoting a more environmentally friendly and comfortable ship environment^18^.

It is well known that Fiber-metal laminates (FMLs), a blended class of materials incorporating the advantages of high strength, high stiffness metallic plies with lightweight, corrossion proof fiber-reinforced polymers. Consequently, FMLs have been extensively researched for various applications in recent years^19–22^. Aluminium alloys are commonly employed as the metallic layers in FMLs^23^. FMLs have found significant applications in vehicle and aircraft surface structures due to their lightweight nature and superior mechanical properties ^24–30^. These structures often encounter acoustic loading from various sources, including flapping caused by loose fasteners, noise from moving parts like tires and engines, and aerodynamic noise^31^. When travelling at high speeds, on differing terrains^32^, the cavity noise caused by the oscillation of air pockets produces a larger band of random noise that can cause acoustic fatigue^33^. An important factor in determining the response of such structures is through the assessment of their vibration response. Such structures fall under the category of multiple degrees of freedom linear systems with characteristic frequency response functions (FRFs), with each FRF illustrating the amplitude and phase of response for a specific degree of freedom^34–38^. The excitation amplitude is linearly inversely proportional to the linear system response. As a result, the FRF develops as a unique characteristic of the system and is independent of the magnitude of excitation. Each FRF has a certain mode shape that can be identified by the modal analysis^39–41^. Low transverse deflection scenarios have been studied using linear dynamic behaviour models, whereas large transverse deflection cases have been studied using non-linear models^42,43^.

Vibration analysis, as a non-destructive method, offers an additional advantage for repeatable investigations on the same samples. In experimental studies on vibration analysis, cantilever beams^44^ as well as simply-supported or clamped-clamped beams^44,45^ have been commonly used. ASTM E756-05 is a widely recognized standard for conducting experiments and determining the loss factor and modulus of uniform, single side damped (Oberst), double side damped (Modified Oberst), and sandwich-type cantilever beams^46^. Researchers have employed various approaches, including analytical, numerical, and experimental methods, to study the vibration damping properties of structural materials^47,48^. Finite element methods based on the first-order shear deformation theory are extensively utilized in both linear and non-linear domains of structural dynamics^39^. McEwan et al.^33^ have observed that the modal behavior of two-dimensional beams subjected to transverse loading is influenced by the spatial distribution and magnitude of the load. The FMLs have been subjected to damping and vibrational characterization as summarized in Table 1.

**Table 1:** Vibration and damping characterization studies conducted on FMLs.

Author	Constituent plies in FMLs	Type of study	Key metrics	Observations
Botelho et al.^24^	AA2024-T3, carbon-fiber/ epoxy, glass-fiber/epoxy	Damping behaviour (<1000Hz Cantilever)	Storage Modulus (E’) and Loss modulus (E”)	Damping of FMLs controlled by dynamic elastic modulus E’
Merzuki et al.^49^	AA2024-T0, woven glass and woven carbon fiber mats	Free Vibration analysis (0-2000 Hz, Cantilever)	Mode shapes and Natural frequency	Natural frequency increased on adding more aluminium layers in FMLs
Xu et al.^50^	Titanium TA2, TC500 Graphite fiber ply with partial constraint layer damping (CLD) patches	Vibration characterization (Cantilever)	Natural frequency, mode shapes, modal damping ratios	Natural frequency and vibration response reduce after employing CLD patches
Li et al.^51^	Ti alloy, ZN33 macromolecule rubber (viscoelastic), E120 carbon fiber with FRD- YG-03 resin	Non-linear vibration behaviour (Cantilever)	Natural frequencies, Damping ratios	multiple viscoelastic layers greatly improve lower order damping performance
Sessner et al.^52^	AA2024-T3, carbon fiber reinforced composite ply, EPDM elastomer plies	Dynamic mechanical analysis (uniaxial tensile mode) and Damping characterization (three-point bending)	Storage modulus ($E_s$), loss modulus ($E_L$) and loss factor (tan($\delta$)) by	Increase in elastomer thickness led to increase in damping, Laminates with aluminium skins showed lower damping coefficient

In order to predict modal frequencies and mode shapes for different material types, an efficient FE model is necessary. This model can then be validated using an impact hammer setup. Pai et al.^53^ have successfully developed such a finite element model to forecast the vibration and damping response of isotropic and homogeneous surface structures. In their study, they focused on AA6061-T6 as the material of interest. The authors conducted experimental tests using impulse hammer excitation and numerical simulations using harmonic analysis. The researchers thoroughly analyzed the impact of factors such as load location, transverse load magnitude, and mesh density on the frequency response functions. This investigation provided detailed insights into the behavior of the vibration response of AA6061-T6.

To assess the capacity of Fiber Metal Laminates (FMLs) to endure vibrations caused by ocean currents on marine vehicle structures, a comprehensive vibration study was conducted on five distinct FML configurations. This investigation specifically focused on hull vibrations on the freedeck (above the waterline) to evaluate their suitability for marine applications. Utilizing impulse hammer excitations, the researchers examined the free-vibration responses of the FMLs, analyzing how the ply arrangements impact vibration performance. The FMLs comprised AA6061-T6 as the surface ply material, with impact-resistant core materials - aramid-epoxy and UHMWPE-epoxy, augmented by an intermediate layer of paperboard to induce acoustic impedance mismatch, a novel approach that enhances vibration damping. A finite element (FE) model was developed to simulate a cantilever beam subjected to harmonically varying transverse loads, mirroring the conditions observed in the experimental study with the impact hammer. The FE results were compared to experimental findings, allowing for an in-depth analysis of how frequency influences the harmonic response across various stackup sequences, ultimately highlighting the potential of FMLs for improved vibration resilience in marine environments.

## Materials and methods

**Fig. 2:**
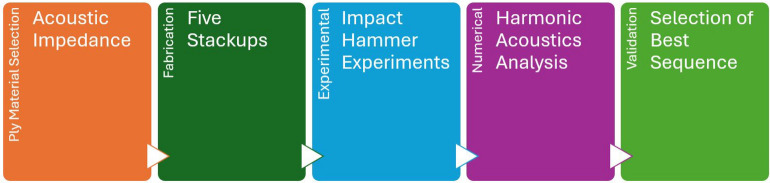
Methodology adopted for the vibration studies on the FMLs.

The Methodology adopted for the current study is shown in Fig. 2. The selection of materials for the FMLs was guided by specific performance criteria essential for marine applications. AA6061-T6, a lightweight yet high-strength aluminum alloy, was chosen as the surface ply due to its excellent strength-to-weight ratio and corrosion resistance, which are critical for long-term durability in marine environments. For the core materials, high-performance synthetic fabrics such as aramid-epoxy and UHMWPE-epoxy were selected to enhance impact resistance, offering superior protection against potential damage from dynamic loads and debris. Additionally, a layer of paperboard was introduced as an intermediate material to create an acoustic impedance mismatch, a novel approach aimed at improving vibration damping. This mismatch between the metal and core layers enhances energy dissipation, effectively reducing vibration amplitude and improving the structural stability of the FMLs under harmonic excitation. Following this, the stackup selection process was carried out, varying the position and number of paperboard plies along with the aramid and UHMWPE plies within the core. By adjusting the number and arrangement of the paperboard layers in each configuration, the study aimed to assess their impact on the stiffness, damping, and vibration behavior of the laminates. The stackups were then fabricated and cut into specimens according to the ASTM E756-05 standard for the impact hammer experiments. Numerical simulations were subsequently performed using the ANSYS Harmonic Analysis tool for each configuration, with the objective of identifying the best-performing sequence among the five.

### Experiments

**Fig. 3:**
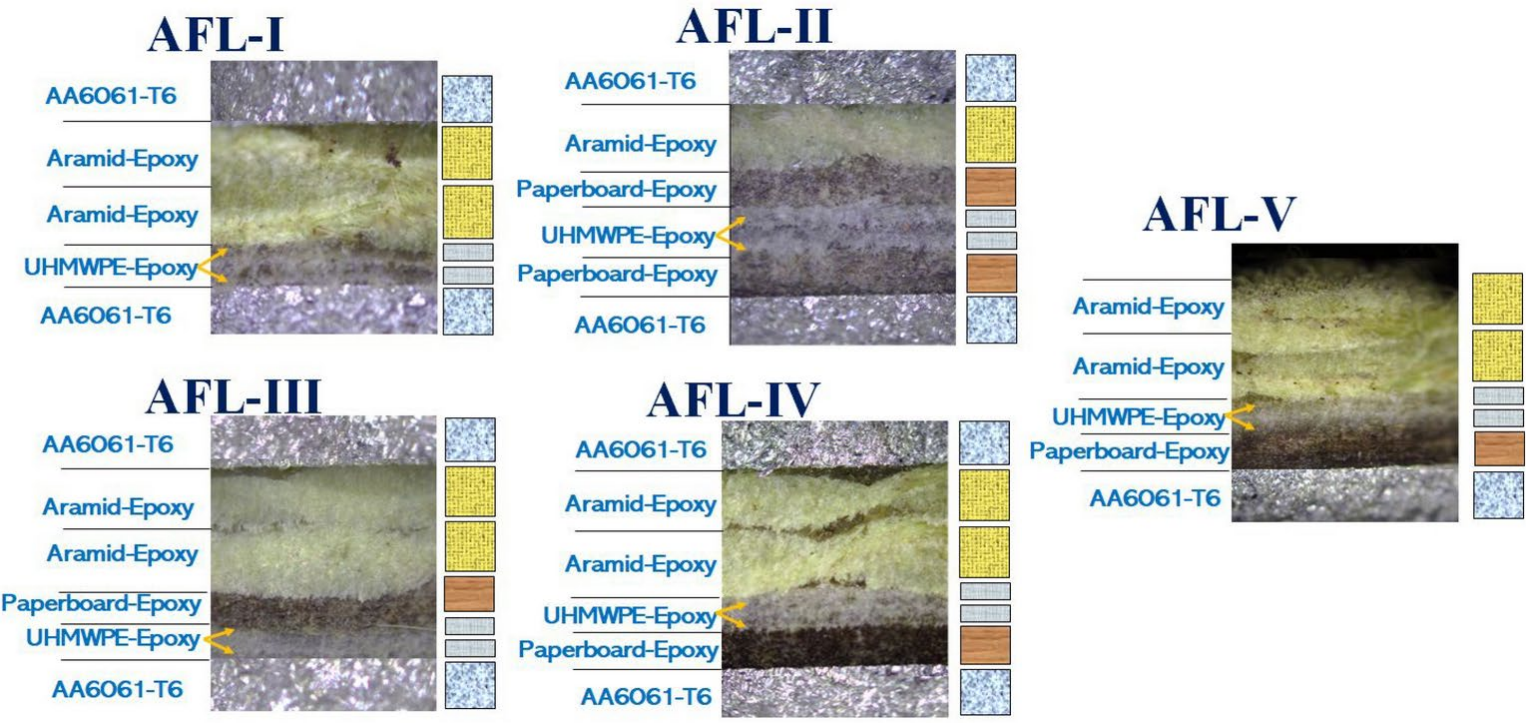
Optical micrographs (cross-sectional) of the layering in the different FML configurations.

For conducting the vibration characterization of the different FML sequences, the impact hammer modal testing was taken up, which is one of the popular techniques^54^. The experimental setup required rectangular test coupons for each of the FML sequences. The details of the five stackups of FMLs considered for the damping studies are shown in Table 2. AA6061-T6 layers of 0.7 mm thickness were used as the surface plies of the laminates. The constituent plies of the core were p-aramid bi-directional fabric (0.3 mm thick, areal density $\sim$ 480 GSM), ultra-high molecular weight polyethylene (UHMWPE) twin-layered, uni-directional fabric (0.25 mm thick, areal density $\sim$ 130 GSM), paperboard (0.3 mm thick, areal density $\sim$ 660 GSM), and epoxy binder. The stackups were fabricated using the compression moulding technique in combination with the hand layup process for stacking the plies. From each stackup, using water jet machining, five test coupons of size $270\,\hbox {mm} \times 25\,\hbox {mm}$ were prepared for the impact hammer experiments. The cross section of the FML sequences were inspected using an Olympus BX53M optical microscope, and the images are shown in Fig. 3.

**Table 2:** Nomenclature of the five sequences of fiber-metal laminates considered for studies.

Sequence	Number of primary layers	Layup details	Overall thickness (mm)	Areal density (kg/m^2^)
AFL-I	6	AA6061/ Aramid-epoxy/ Aramid-epoxy/ UHMWPE-epoxy/UHMWPE-epoxy/ AA6061	3.43	5.43
AFL-II	7	AA6061/ Aramid-epoxy/ Paperboard-epoxy/ UHMWPE-epoxy/ UHMWPE-epoxy/ Paperboard-epoxy/ AA6061	3.75	6.55
AFL-III	7	AA6061/ Aramid-epoxy/ Aramid-epoxy/ Paperboard-epoxy/ UHMWPE-epoxy/UHMWPE-epoxy/ AA6061	3.46	6.27
AFL-IV	7	AA6061/ Aramid-epoxy/ Aramid-epoxy/ UHMWPE-epoxy/ UHMWPE-epoxy/ Paperboard-epoxy/ AA6061	3.50	6.30
AFL-V	6	Aramid-epoxy/ Aramid-epoxy/ UHMWPE-epoxy/ UHMWPE-epoxy/ Paperboard-epoxy/ AA6061	2.85	4.76

**Fig. 4:**
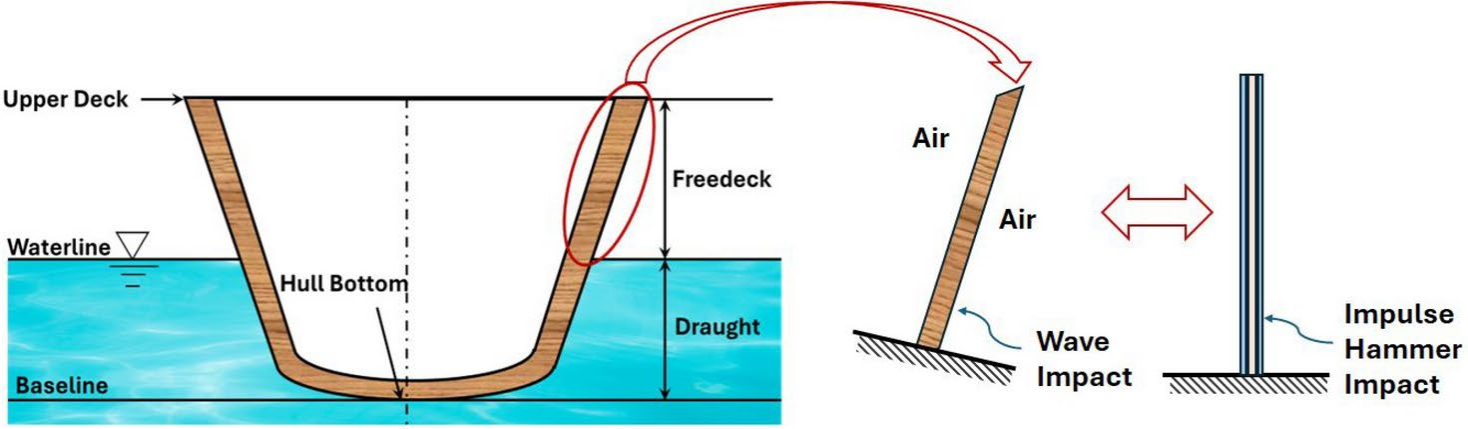
Numerical model formulation representing wave impact on the ship hull side.

**Fig. 5:**
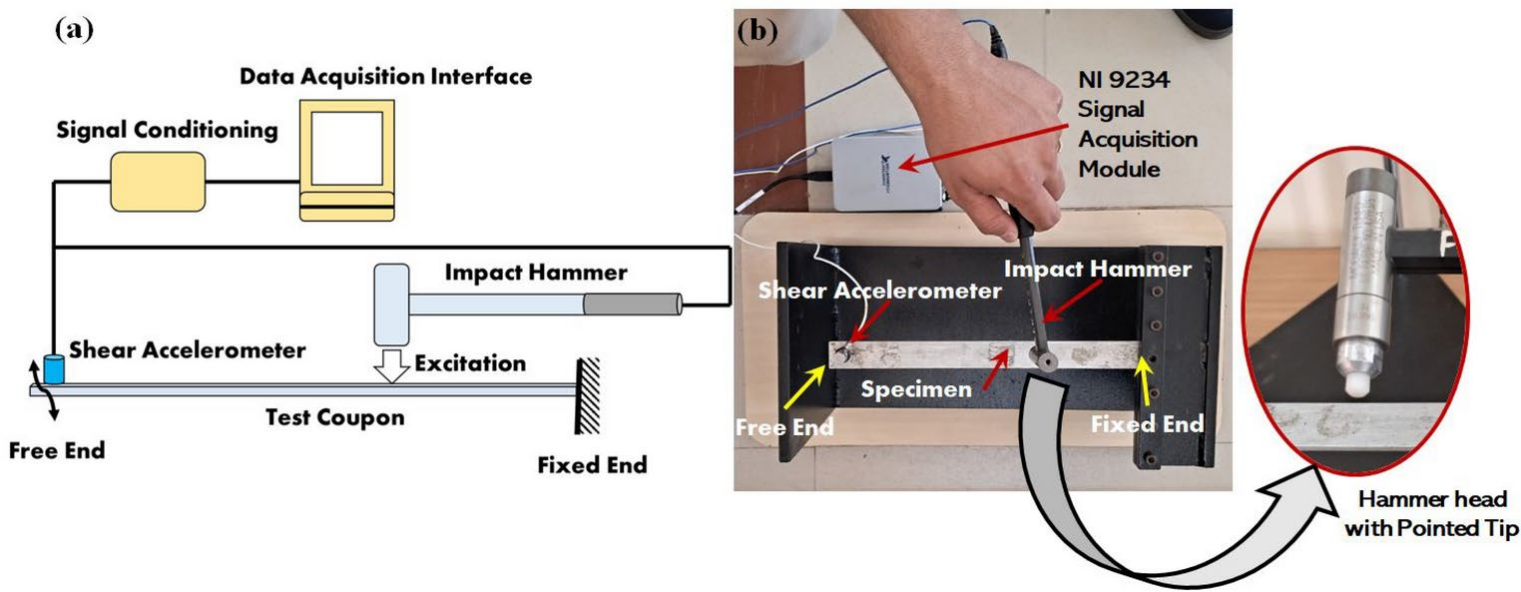
(**a**) Schematic of the impact hammer excitation setup (**b**) Experimental setup with specimen being excited by the impact hammer.

The dynamic loads and boundary conditions encountered by the ship hull on the freedeck side were analogized to those experienced by a cantilever beam subjected to excitation near its clamping area as shown in Fig. 4. According to the principles of naval architecture, for side plating design, the plating should be 70 times the thickness, to prevent buckling^55,56^. Hence, to ensure geometric similarities between the FE model and the marine structure, an average slenderness ratio (ratio of plate span to thickness) of $\sim$ 74:1, was taken up in the current study. The experimental model focuses on a section of the hull that may represent areas from the bow, beam, or quarter on either the starboard or port side. The beam vibration w.r.t. the support replicates the oscillation of the ship hull around the waterline on the port or starboard side. To capture this effect, a case with a 90-degree inclination was incorporated. Hence, during impulse hammer experiments, 270 mm long test coupons were taken up. During the experiment, each coupon was clamped at one end and the accelerometer was fitted at the free end as shown in Fig. 5, with 250 mm as the overhanging length (Fixed-Free boundary condition). For the different sequences, the slenderness ratio varied between 67:1 for AFL-V to 88:1 for AFL-II. The shear accelerometer (Make: PCB 352C36, Sensitivity: 101.6 mV/g) was connected to the signal conditioning amplifier and the data acquisition/ user interface (Make: NI 9234) to record the data from the excitation by an impulse hammer (Make: PCB 086C03). The impulse hammer excitations were carried out on the specimens at a location 60 mm from the supports, as per ASTM E756-05 standard^46^. The amplitude-frequency plots were obtained in the frequency range of 0-500 Hz^57^ for five specimens from each FML sequence. The material modulus was computed using Eq. (1). The modal coefficients $\hbox {C}_n$ were obtained from Table 3. The shear accelerometer was weighed for accomodating a point mass in the finite element model. The accelerometer mass was measured to be 4.8 g.

1 $$\begin{aligned} E_s=\frac{12\rho L^4{f_n}^2}{t^2{C_n}^2} \end{aligned}$$

**Table 3:** Values of the modal coefficients at different modes^46^.

Mode number ‘*n*’	$\hbox {C}_n$
1	0.55959
2	3.5069
3	9.8194
4	19.242
5	31.809
*n*	$\frac{\pi (n-0.5)^2}{2}$ for $n>3$

### Numerical model

**Fig. 6:**
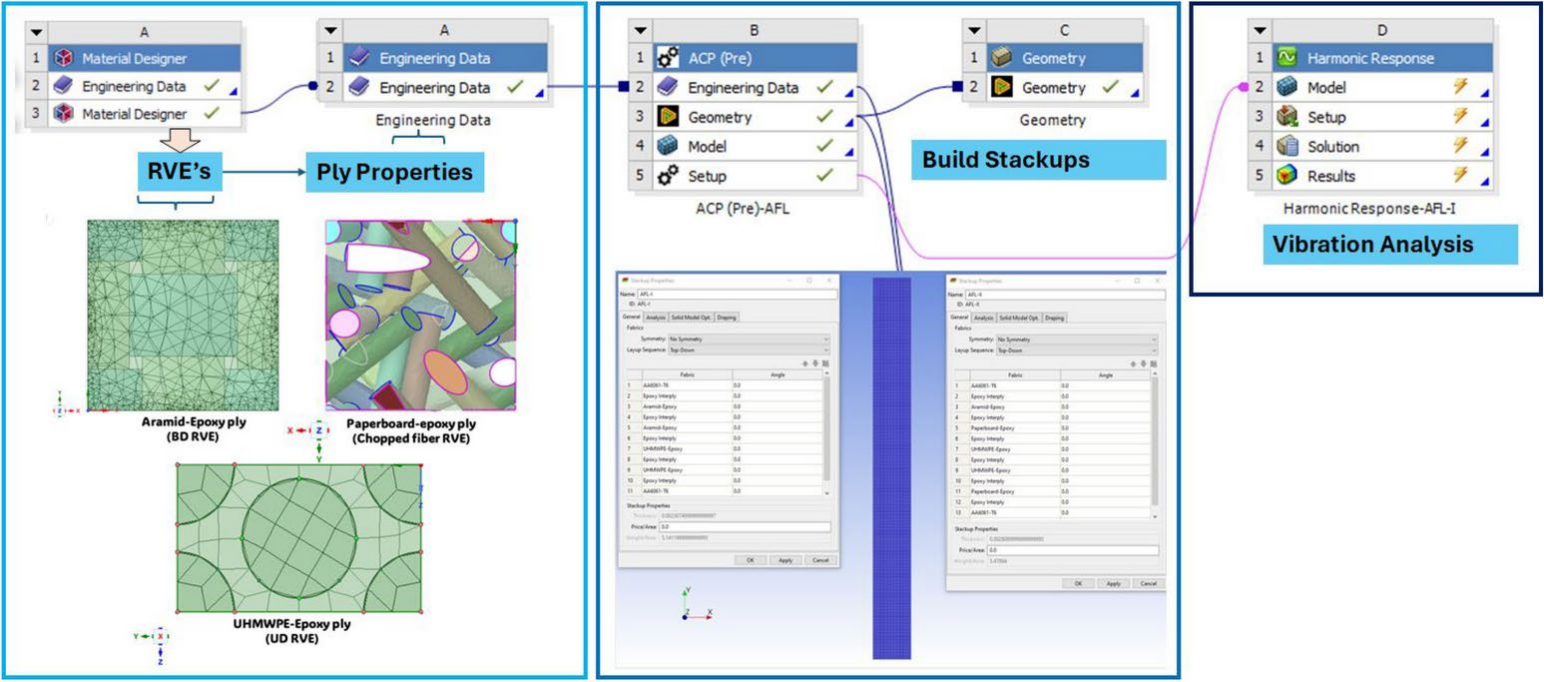
Different tools of ANSYS FE software used for the numerical analysis- material designer, ACP-Pre, and harmonic analysis.

**Fig. 7:**
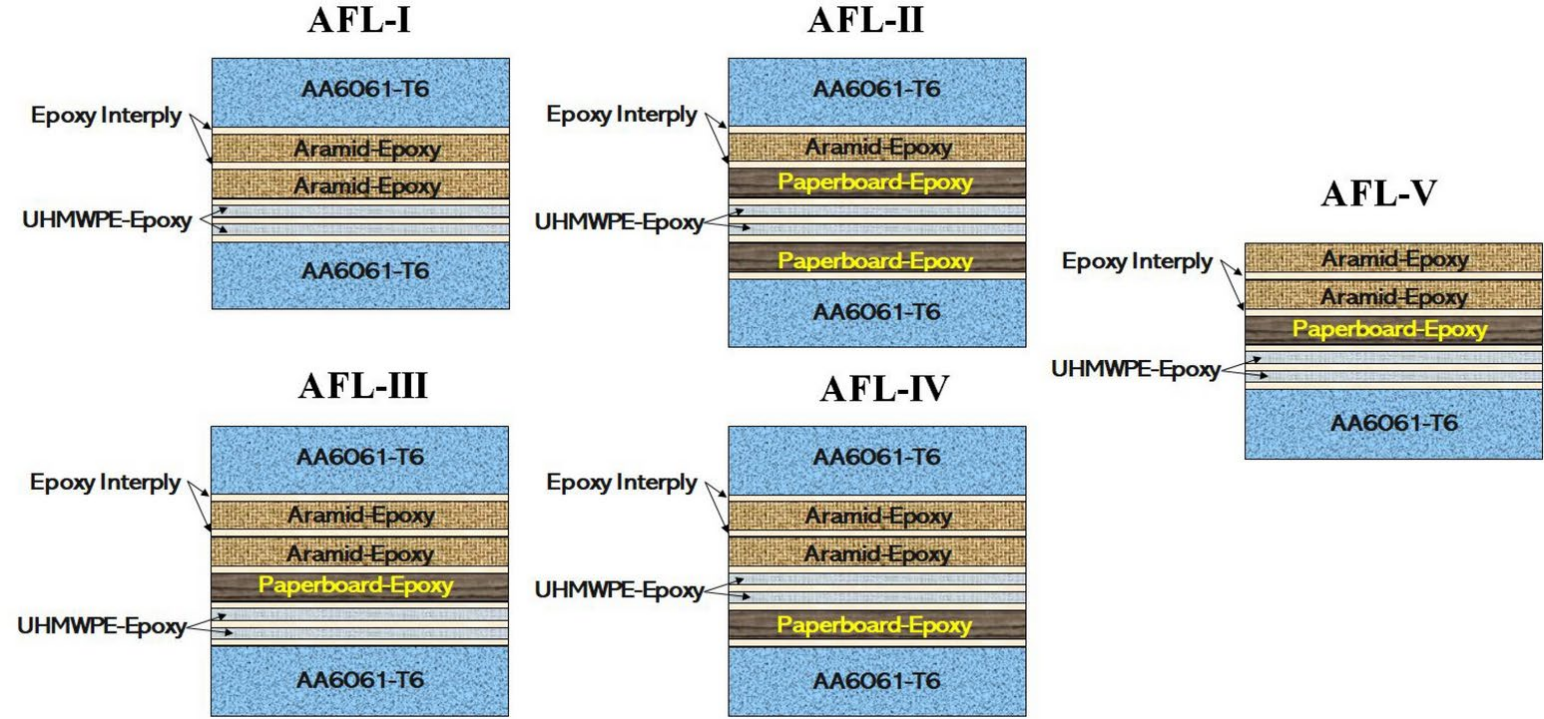
Stackup sequence of the FMLs with a thin Epoxy interply.

The numerical analysis was taken up using different tools of ANSYS finite element software as shown in Fig. 6. For obtaining the properties of the core layers (aramid-epoxy, UHMWPE-epoxy and paperboard-epoxy), ANSYS Material Designer module was used. The obtained properties are displayed in Table 4 which were assigned to the respective plies in the stackups. The different Representative Volume Elements (RVEs) are shown in Fig. 6. Each stackup was modeled on ANSYS ACP-Pre tool, with the ply sequence as shown in Fig. 7. The optical micrographs of the cross-section highlighted the need for a thin epoxy interply (0.02 mm thick) between each set of primary plies to account for the binder at the interface. The laminate data from ANSYS ACP-Pre was then transferred to the Harmonic Response tool, for vibration analysis.

**Fig. 8:**
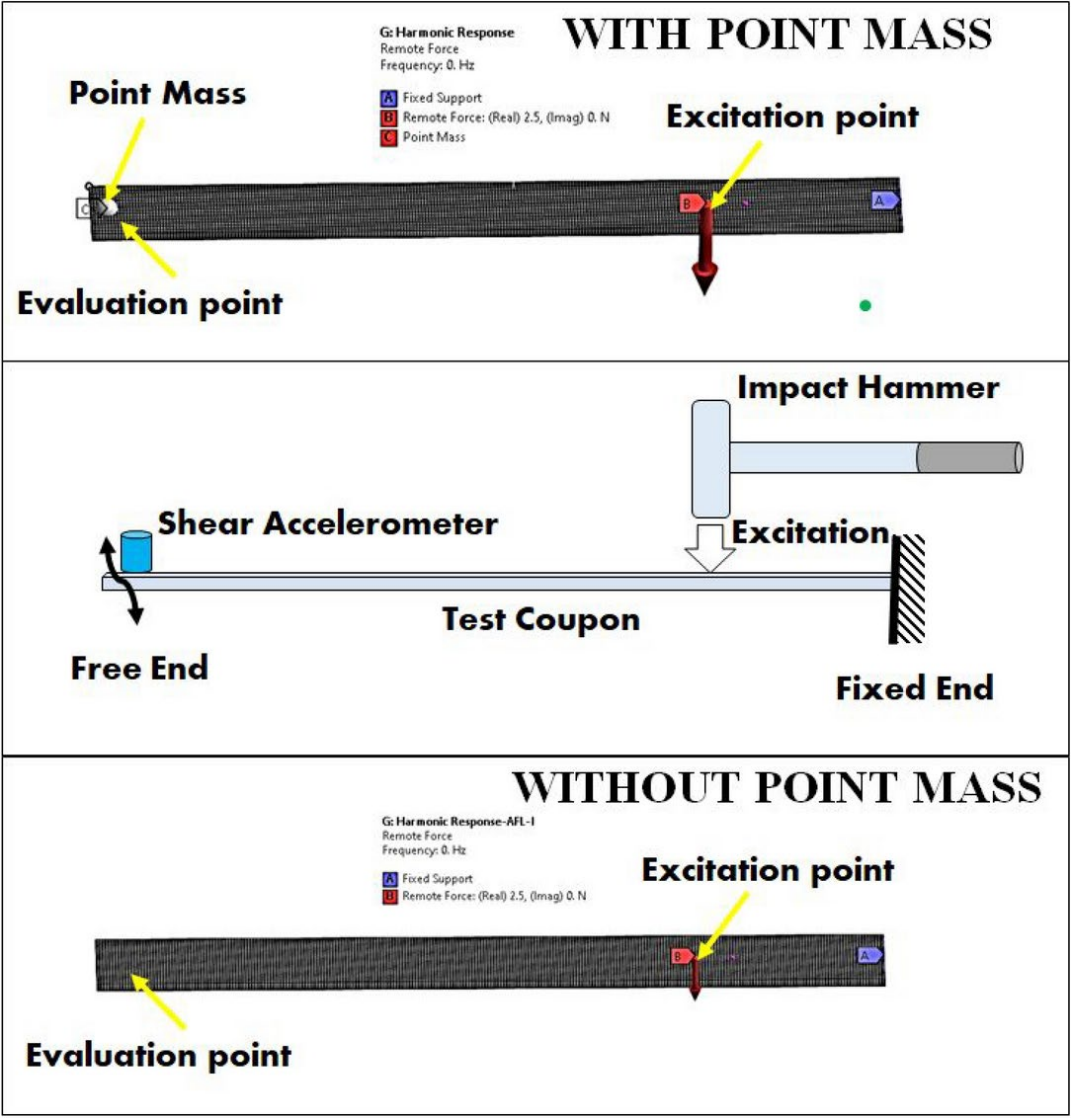
Numerical FE models for harmonic analysis of the sequences with and without point-mass considerations.

**Table 4:** Ply properties for different layers used in the sequences.

Properties	AA6061-T6	Aramid-epoxy	UHMWPE-epoxy	Paperboard-epoxy	Epoxy interply
Density ($kg/m^3$)	2760	1380	1210	1250	1186
$E_{1}$ (GPa)	69	13.7	3.23	3.95	3.01
$E_{2}$ (GPa)	69	13.7	3.24	3.86	3.01
$E_{3}$ (GPa)	69	4.96	3.01	4.06	3.01
$\nu _{12}$ (GPa)	0.33	0.52	0.37	0.304	0.35
$\nu _{23}$ (GPa)	0.33	0.263	0.41	0.197	0.35
$\nu _{31}$ (GPa)	0.33	0.263	0.37	0.226	0.35
$G_{12}$ (GPa)	25.9	4.84	1.1	1.51	1.40
$G_{23}$ (GPa)	25.9	2.91	1.07	1.50	1.40
$G_{31}$ (GPa)	25.9	2.91	1.09	1.57	1.40

For vibration analysis, the FE model was setup in line with the experiments undertaken with the impulse hammer. The test coupon geometry was replicated in the numerical model, with a point of mass of 4.8 g placed near the free end as shown in Fig. 8. An alternative model without the point mass was also considered to measure the influence of the point mass on the vibration response of the FMLs as shown in Fig. 8. The multizone tri/quad meshing approach with unit skewness was assigned for the mesh characteristics. A mesh size of 1 mm was considered based on the grid independence check for mesh sizes 0.25 mm - 5 mm using first natural frequency ’$\hbox {f}_n$’ as the parameter. This beam featured a transverse, harmonically varying load with a magnitude approximately equal to the weight of the impulse hammer head ($\sim$ 2.5 N), acting at a distance of 60 mm from the fixed end. At the response location (accelerometer position), a point on the geometry was selected as the evaluation point. At this point, The acceleration-frequency response functions were extracted, the frequency spacing was taken to be linear, with mode superposition solution method. The results from the simulations for the stackups were then compared with those obtained from the experimental results.

## Results and discussion

**Fig. 9:**
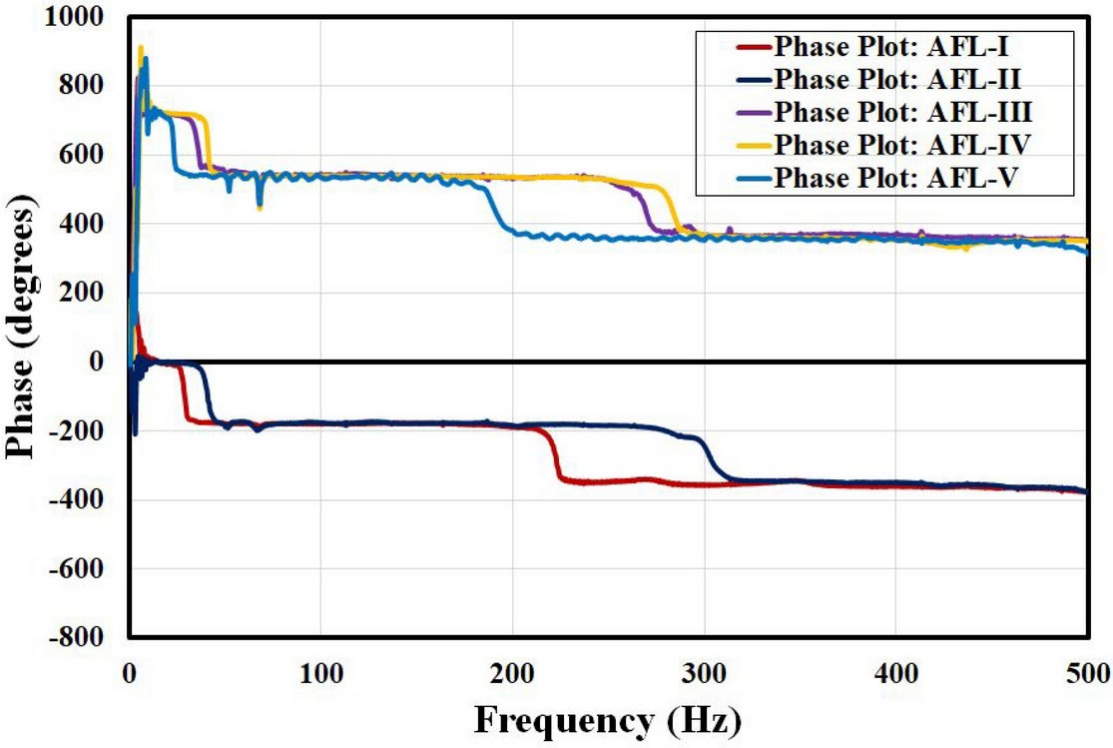
Phase-frequency plots for the different sequences.

The data from the impact hammer experiments were analyzed to derive the frequency response functions (FRFs) and phase-frequency plots. Phase-frequency plots, commonly referred to as Bode plots, are widely employed in vibration analysis to visually depict the interplay between the phase and frequency of a vibrating system. These plots offer valuable insights into the dynamic behavior relative to the natural frequencies and associated vibration modes^14^. The phase plots for the different sequences are shown in Fig. 9. The observed phase dips correspond to the natural frequencies identified in the various sequences.

**Fig. 10:**
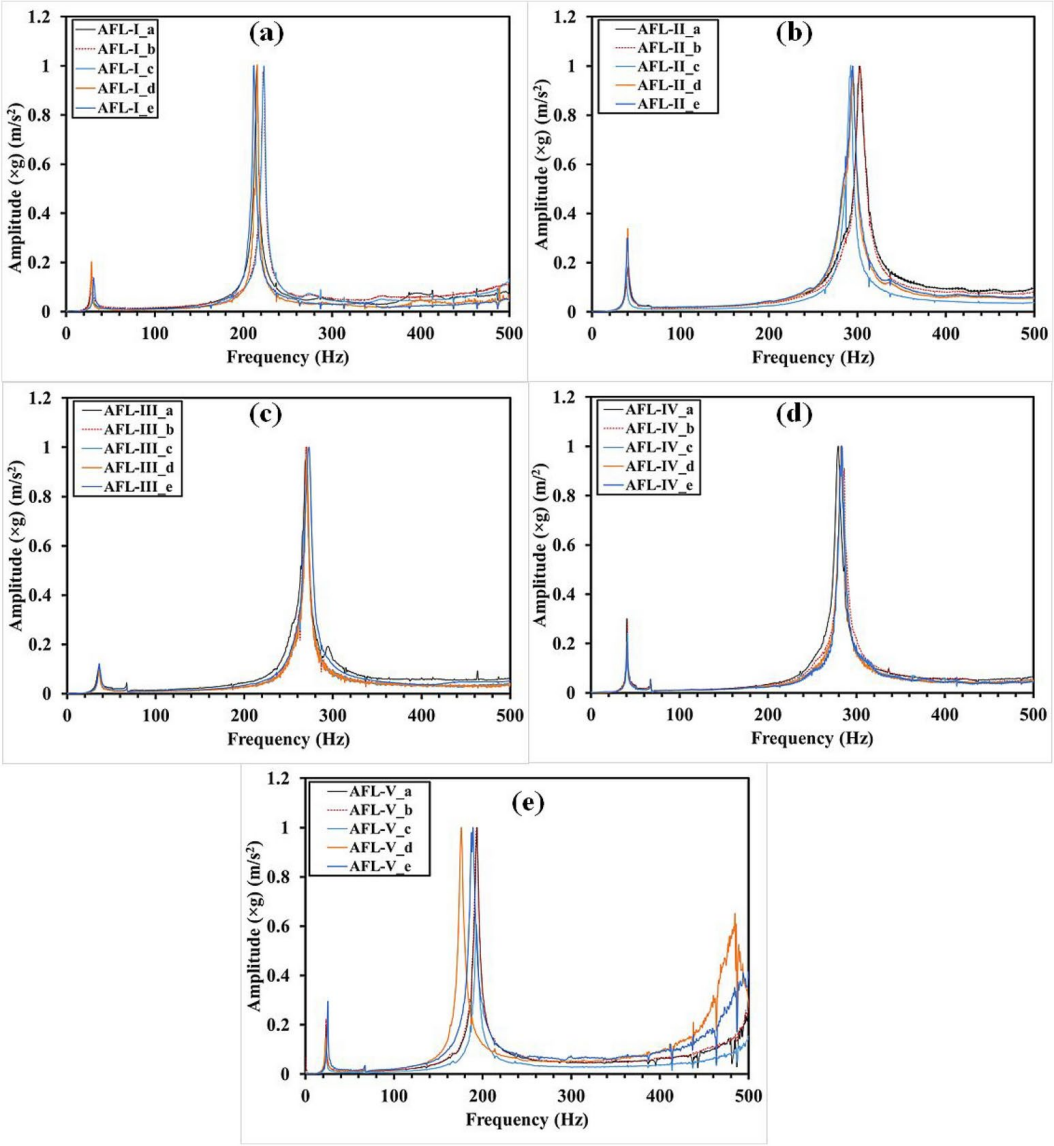
Frequency response functions derived from the impulse hammer experiments for the FML stacking sequences.

**Fig. 11:**
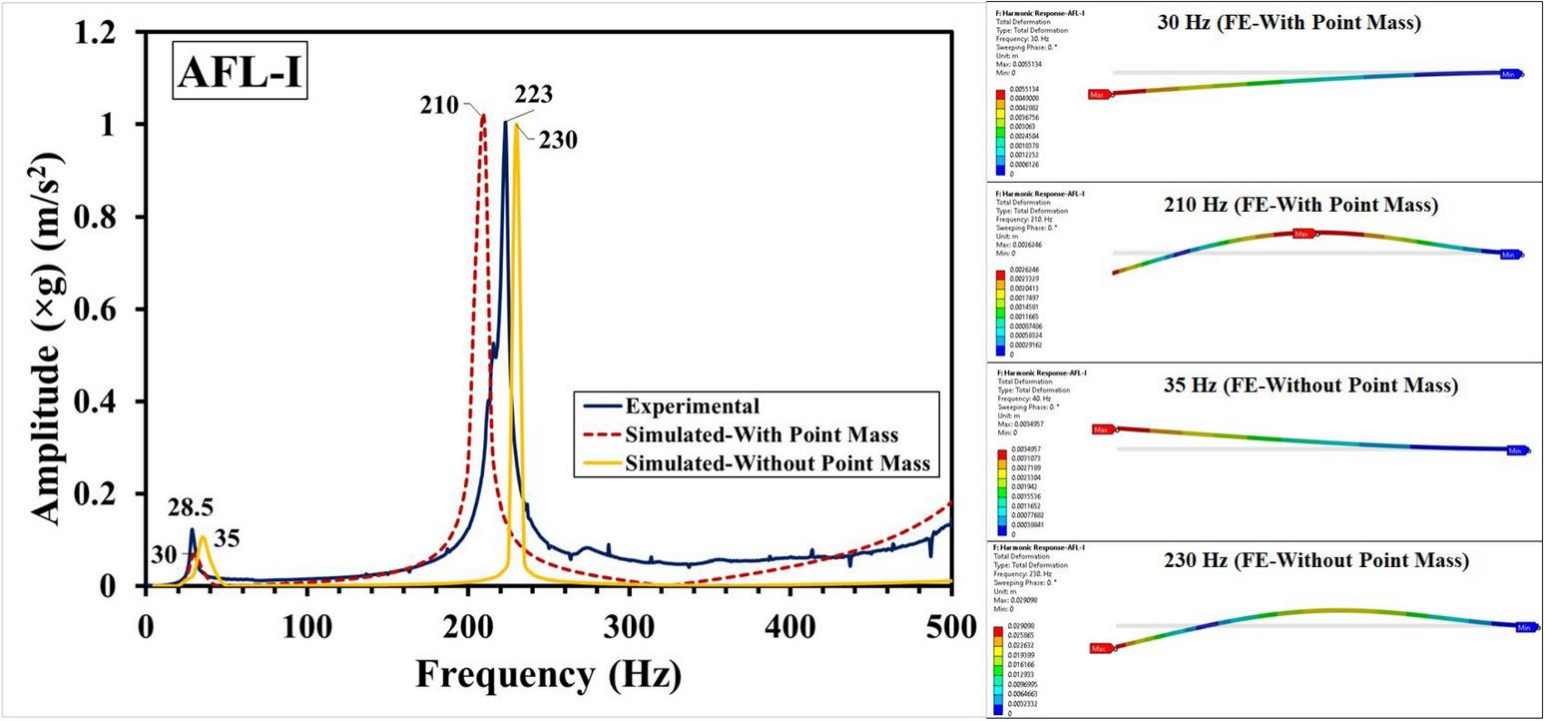
Frequency response functions for AFL-I comprising experimental, FE-With point mass and FE-without point mass consideration, and mode shapes.

**Fig. 12:**
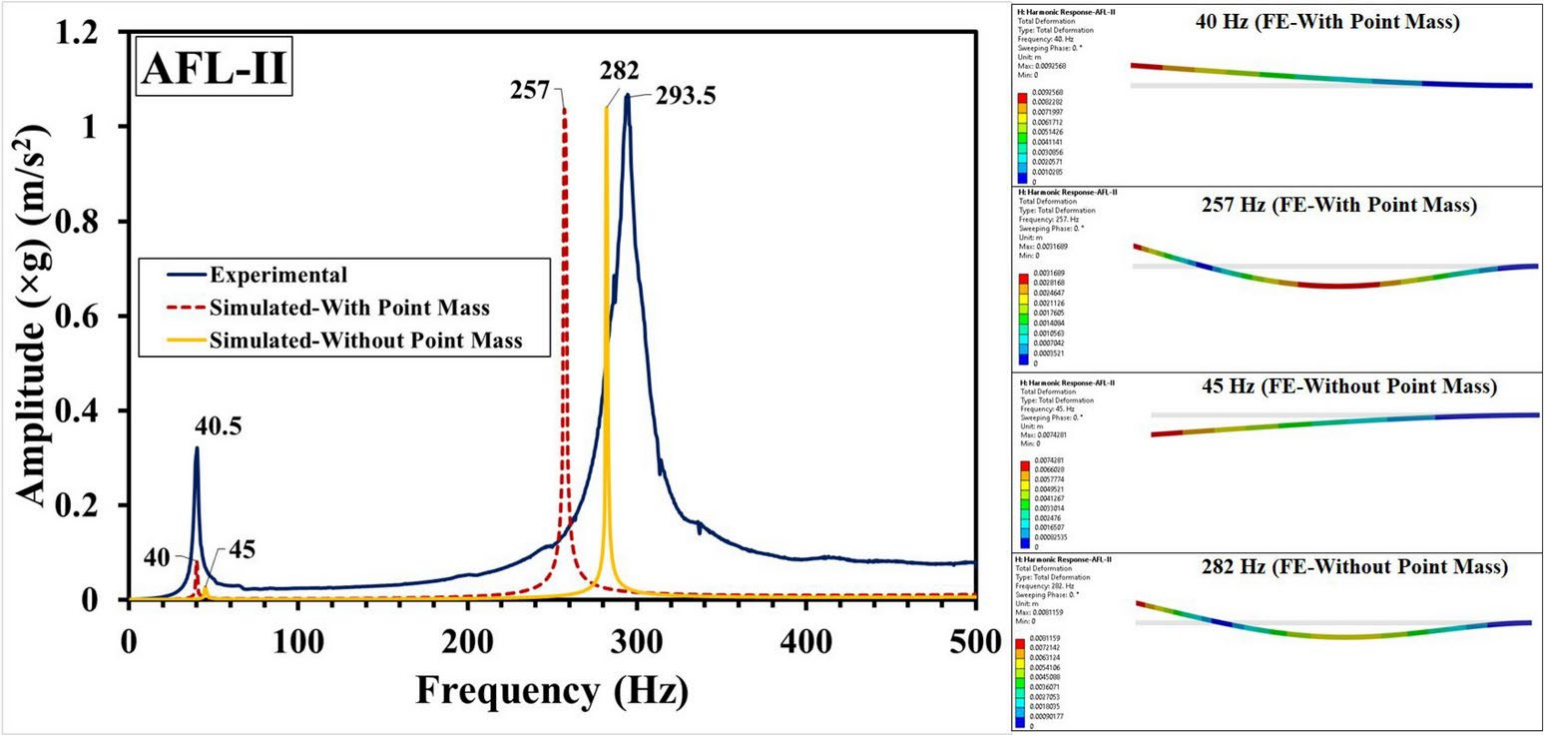
Frequency response functions for AFL-II comprising experimental, FE-with point mass and FE-without point mass consideration, and mode shapes.

**Fig. 13:**
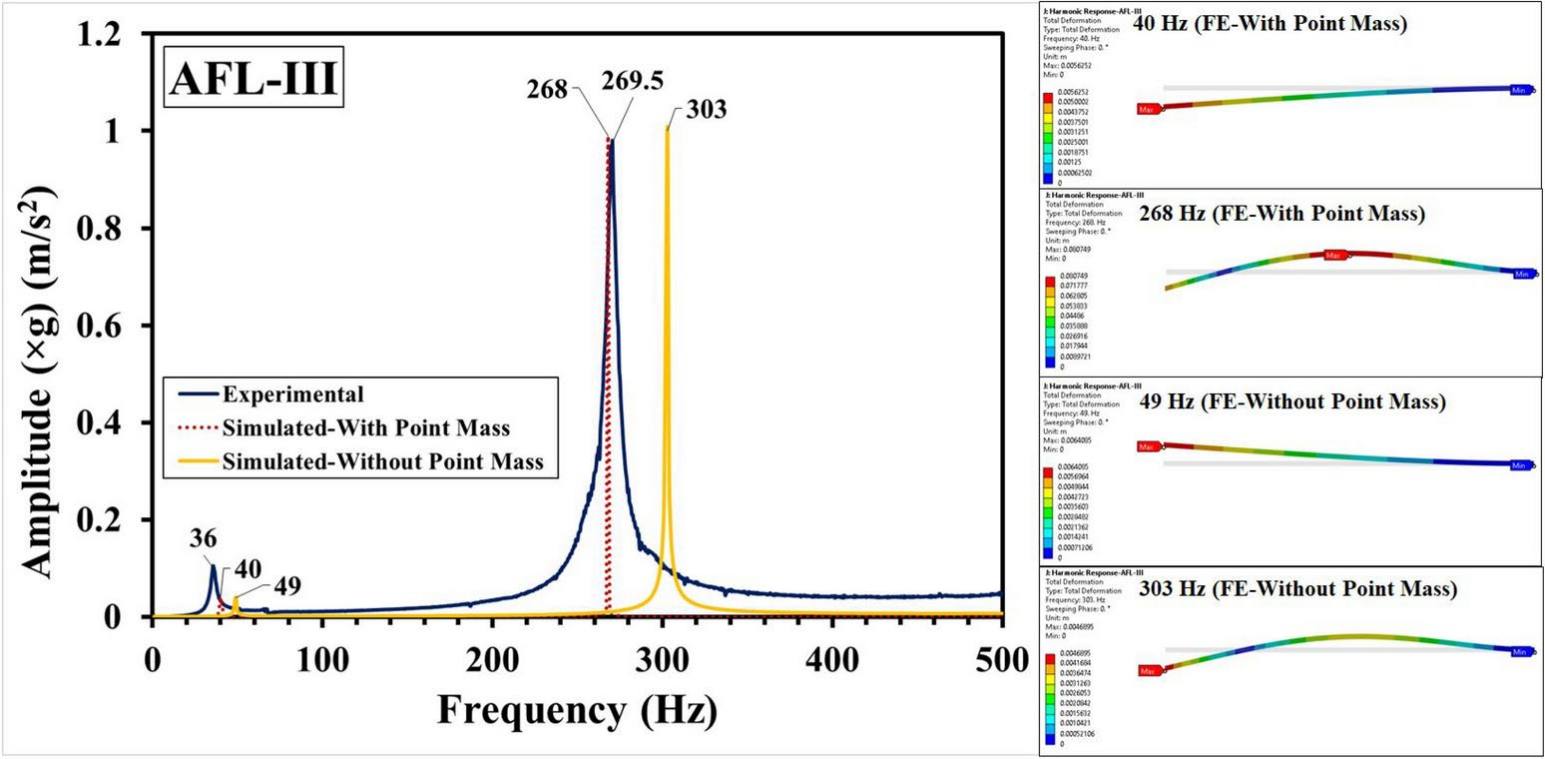
Frequency response functions for AFL-III comprising experimental, FE-with point mass and FE-without point mass consideration, and mode shapes.

**Fig. 14:**
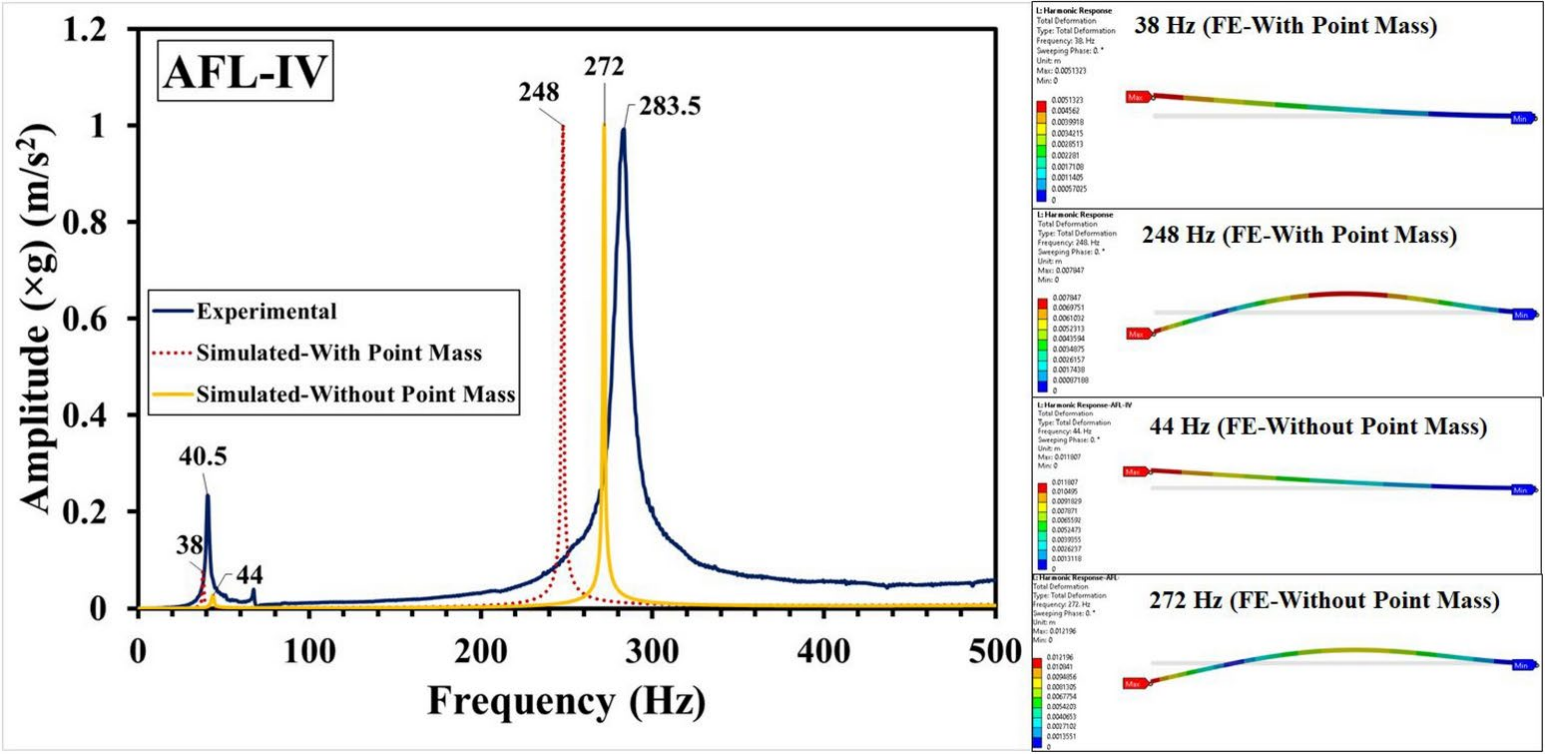
Frequency response functions for AFL-IV comprising experimental, FE-with point mass and FE-without point mass consideration, and mode shapes.

**Fig. 15:**
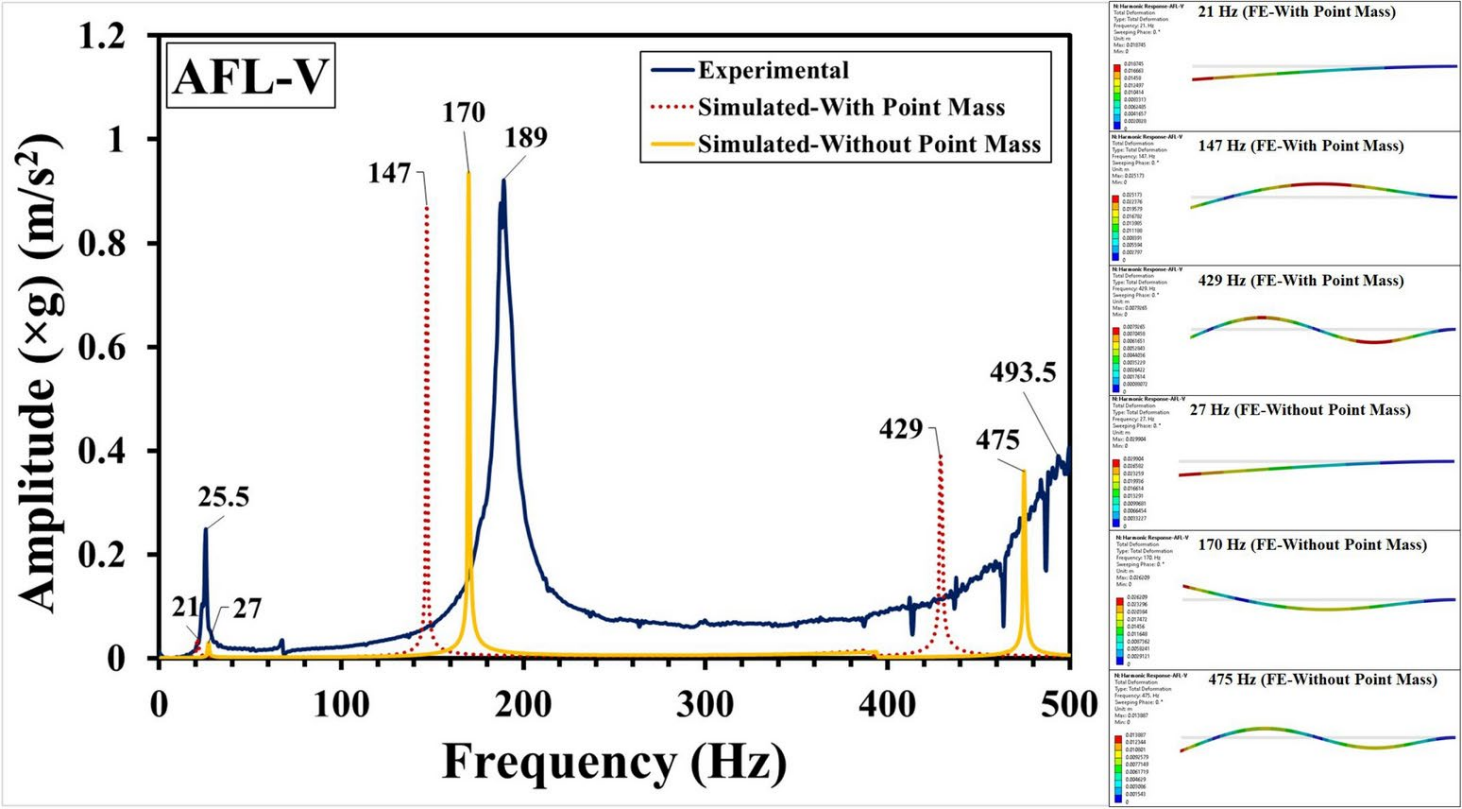
Frequency response functions for AFL-V comprising experimental, FE-with point mass and FE-without point mass consideration, and mode shapes.

**Table 5:** Sequence-wise comparison of modal frequencies from numerical and experimental results.

Sequence	Mode number (*n*)	Natural frequency ($f_n$) (Hz)		
		Experimental (average)	FE-with point mass	FE-without point mass
AFL-I	1	29.3 ± 1.86	30	35
	2	217.7 ± 9.14	210	230
AFL-II	1	40.4 ± 2.04	40	45
	2	297.5 ± 8.79	257	282.5
AFL-III	1	36 ± 0.90	40	49
	2	270.7 ± 2.06	268	303
AFL-IV	1	40.7 ± 0.49	38	44
	2	282.6 ± 3.87	248	272
AFL-V	1	23.3 ± 0.49	21	27
	2	180.4 ± 6.53	147	170
	3	495.1 ± 5.66	429	475

The FRFs are shown in Fig. 10 for the different sequences respectively. For the sequences AFL-I, AFL-II, AFL-III, and AFL-IV, two modes and their corresponding natural frequencies were observed. In contrast, AFL-V exhibited three modes, with the respective natural frequencies. When comparing the results of AFL-I and AFL-II, the addition of two paperboard-epoxy layers within the stacking sequence, along with the removal of one aramid-epoxy ply, led to a clear shift in the mode 2 frequency to the right. This suggests that the mass effect outweighed the stiffness effect, broadening the range for mode 2 frequencies. In AFL-III, the key difference from AFL-I was the placement of a paperboard-epoxy ply centrally, just before the final UHMWPE-epoxy layers. While the mode 1 frequency stayed relatively unchanged, a modest rightward shift in the mode 2 frequency was noted. AFL-IV followed a similar trend for mode 1 when compared to AFL-I, but with the paperboard-epoxy ply positioned as the penultimate layer, it produced a more pronounced shift in mode 2 than AFL-III. AFL-V sequence was similar to AFL-IV, but without the AA6061-T6 faceplate. The absence of this high-stiffness faceplate caused a significant leftward shift in mode 2 frequency and introduced a third mode frequency as a result of this shift. When the paperboard-epoxy ply was incorporated alongside the aramid-epoxy and UHMWPE-epoxy layers in AFL-II, AFL-III, and AFL-IV, a clear upward shift in natural frequencies was observed. This shift can be linked to the damping properties, enhanced energy dissipation, and the mismatch in acoustic impedance caused by the lower acoustic impedance of the paperboard. Additionally, AFL-II and AFL-IV showed a substantial increase in acceleration amplitude for mode 1 compared to AFL-I and AFL-III. In AFL-V, all three modes experienced a leftward frequency shift, driven by the absence of the high-stiffness AA6061-T6 faceply, which notably reduced the overall stiffness, allowing the mass to dominate and influence the frequency response.

The experimental and numerical results have been compared in Fig. 11 for the sequence AFL-I, Fig. 12 for the sequence AFL-II, Fig. 13 for the sequence AFL-III, Fig. 14 for the sequence AFL-IV, and Fig. 15 for the sequence AFL-V respectively. Alongside the FRFs, the mode shapes for the respective modes 1,2 (AFL-I to AFL-IV) and modes 1, 2 and 3 (AFL-V) are also shown. For each of the stackups, two frequency response functions (FRFs) were generated by the numerical model: one considering the point mass of 4.8 g and the other without it. Incorporating the point mass in the finite element model resulted in a reduction in both mode 1 and mode 2 frequencies. In AFL-I, the FE results that included the point mass closely matched the experimental data, with less than 6% error for mode 1. In contrast, the results without point mass consideration showed a much larger error of 22.8%. For mode 2, the error with point mass consideration was 5.8%, while the error without it was slightly lower at 3.2%. In AFL-II, the mode 1 frequency from the FE model with point mass consideration aligned even more closely with the experimental results, showing only a 1.2% error. Without the point mass, the error increased to about 11%. Interestingly, for mode 2, the model with point mass consideration produced a higher error of 12.4%, while the model without it showed a smaller error of around 4%. For AFL-III, the mode 1 frequency from the FE model with point mass consideration had a moderate agreement with the experiment, with an error of approximately 11%. The model without point mass consideration, however, had a much higher error of about 26%. For mode 2 in AFL-III, including the point mass led to a remarkably accurate result with less than 1% error, while excluding the point mass resulted in a larger error of around 12.5%. AFL-IV followed a similar trend. For mode 1, the point mass model had an error of around 6%, while the model without the point mass had a slightly higher error of 8.7%. In mode 2, the error increased to 12.5% when the point mass was included, compared to 4% without it. In AFL-V, both point-mass and no-point-mass FE models showed the third modal frequency, confirming the leftward shift caused by the absence of the AA6061-T6 faceplate. For mode 1, the model with point mass consideration had a much larger error of 17%, while without the point mass, the error was reduced to around 5.8%. For mode 2, the point mass model showed a significant 22.22% error, whereas excluding the point mass led to a smaller error of approximately 10%. The natural frequencies for the respective modes in case of the different sequences has been compared in Table 5. Including the point mass in the numerical model consistently led to a reduction in the mode frequencies across all configurations. This adjustment significantly improved the accuracy of the results, especially for mode 1, where the FE model closely matched the experimental data. However, variations between the FE and experimental results can still be traced to a few experimental factors, such as slight inconsistencies in the hammer impact locations, varying force intensities, or even minor changes in the condition of the accelerometer connections. Additionally, real-world variations in layer properties, like inhomogeneities and bond strength at the ply interfaces - particularly between the AA6061-T6 and FRP layers - could also introduce discrepancies. In the FE model, assumptions such as perfectly bonded ply interfaces and using an orthotropic material model for the intermediate plies likely contributed to the remaining deviations. Despite these complexities, incorporating the point mass in the FE analysis significantly reduced the error, bringing the numerical modal frequencies much closer to the experimental values across different stacking sequences. This demonstrates the critical influence of mass consideration on the dynamic behavior of these FMLs. As an extension of the error analysis of the two finite element approaches against the experimental data, the Root Mean Square error was calculated as given by Eq. (2). This metric provides a quantitative measure of the average deviation between the predicted modal frequencies from each FE model and the observed experimental frequencies. The RMS error was computed for the natural frequency $f_n$ values from numerical simulation with respect to those values obtained form the experimental results, with reference to Table 5. The values of the RMS error “$\epsilon _{rms}$” are shown in Table 6. The RMS error for Mode 1 was least (1.08 %) for the FE-With Point Mass consideration, while for the FE-Without Point Mass consideration, it was 3.14 %. Likewise, for Mode 2, the least error (8.1 %) was observed for the FE-Without Point Mass consideration, while for the FE-With Point Mass consideration, the error was 12.68 %. Since Mode 3 was observed for the sequence AFL-V, the RMS error for the FE-With Point Mass was 66.1 %, while for the FE-Without Point Mass, the error was 20.1 %. For all the modes, across the five sequences, the overall RMS error for FE-With Point Mass was 27.66 %, while that for the FE-Without Point Mass consideration was 14.43 %. Therefore, while the FE model with point mass consideration proved optimal for predicting Mode 1 frequency, the FE model without point mass consideration demonstrated greater accuracy for higher modal frequencies.

2 $$\begin{aligned} \epsilon _{rms} = \sqrt{\frac{1}{N} \sum _{i=1}^{N} (y_i - \hat{y}_i)^2} \end{aligned}$$

Where: $N$ is the total number of data points, $y_i$ is the observed frequency (Hz) (experimental data) for the $i$-th mode, $\hat{y}_i$ is the computed frequency value (from FE model simulation results) for the $i$-th mode.

**Table 6:** RMS error analysis for the FE-with point mass and FE-without point mass considerations.

Mode number (*n*)	RMS error ($\epsilon _{rms}$) (Hz)	
	FE-with point mass	FE-without point mass
1	1.08	3.14
2	12.68	8.1
3	66.1	20.1
Overall	27.66	14.43

**Fig. 16:**
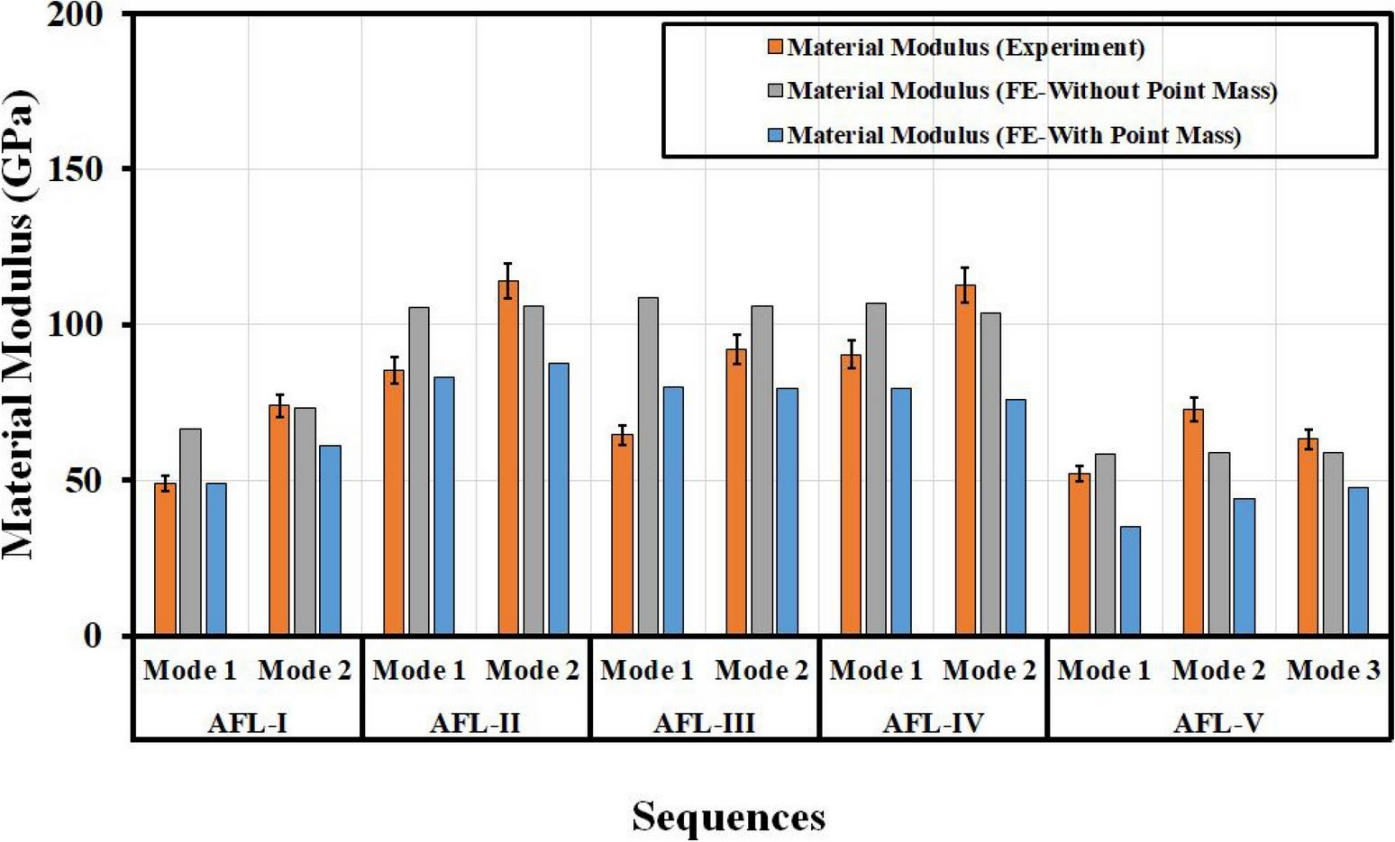
Comparison of material modulus for different sequences found by numerical and experimental approaches.

The material modulii for the various sequences were computed from Eq. (1) for the numerical and experimental results. The values have been shown in Fig. 16. The Mode 1 moduli from the numerical analysis with point mass consideration closely aligned with the experimental results, while the Mode 2 moduli showed slightly less accuracy. However, as seen with the frequency predictions, the FE analysis without point mass consideration tended to overestimate the material moduli across the various stacking sequences. When analyzing the material response of the fiber metal laminates (FMLs), AFL-II and AFL-IV exhibited the highest moduli, indicating superior stiffness compared to the other sequences, with AFL-III following closely behind. AFL-I demonstrated greater stiffness than AFL-V, underscoring the significant impact of the AA6061-T6 metallic face ply on the overall rigidity of the laminates. Based on these findings, AFL-II and AFL-IV stand out as the most optimal sequences in terms of material stiffness among the five configurations.

## Conclusions

This work focused on investigating the vibration response of multi-layered fiber metal laminates in the marine vehicle structural applications. The laminates were composed of AA6061-T6 skins, high-performance fiber-reinforced plies made of aramid-epoxy and UHMWPE-epoxy, and paperboard plies, which were introduced to create an acoustic impedance mismatch at the inter-ply regions. The vibration and damping characteristics of these laminates were assessed through impact hammer experiments and finite element numerical simulations. The frequency response functions of the multi-layered laminates, incorporating both metallic and fiber-reinforced polymer plies, were thoroughly analyzed using the developed finite element model with and without the point mass consideration. From these studies, the following conclusions were drawn:

- Four FML sequences (AFL-I to AFL-IV) exhibited 2 modes, while AFL-V showed 3 modes. The absence of the AA6061-T6 metallic face ply in AFL-V significantly reduced the overall stiffness, leading to lower modal frequencies and reduced modulus values. Hence, inclusion of metallic face ply in fiber metal laminates (FMLs) is important for applications that require effective vibration damping and high stiffness.
- Incorporating a paperboard-epoxy ply between the layers of the fiber metal laminate (FML) sequences resulted in an increase in modal frequencies. This observation underscores the significance of impedance mismatch in enhancing the damping characteristics of FMLs, suggesting that the strategic placement of such materials can effectively influence their dynamic behavior.
- The inclusion of point mass consideration in the numerical model was pivotal in achieving accurate predictions of mode frequencies, particularly for Mode 1, where the results closely matched experimental findings.
- However, for Mode 2, the FE analysis without point mass consideration demonstrated greater accuracy, indicating that the point mass consideration may not always be beneficial for higher modes.
- To further compare the modal frequency predictions by the FE models, the higher RMS error for the FE model with point mass consideration, compared to that without point mass consideration, indicated that the FE model without point mass consideration was more accurate in predicting the higher modal frequencies across the FML configurations.

The results revealed that AFL-II and AFL-IV not only exhibited the highest material moduli but also demonstrated advantageous frequency response characteristics, highlighting their robustness in dynamic applications. The specific layering of plies in these sequences contributed to their superior performance, suggesting that these configurations are particularly well-suited for applications demanding exceptional vibration damping and structural integrity. This makes them promising candidates for environments where reliability and performance are critical, such as marine structures.

## Data Availability

The authors declare that the data supporting the findings of this study are available within the paper.
